# The participation of hard-to-reach older people in the research and development process of health technologies from the perspective of multipliers—A qualitative analysis

**DOI:** 10.3389/fpubh.2024.1334180

**Published:** 2024-06-03

**Authors:** Alexander Pauls, Frauke Koppelin, Hajo Zeeb

**Affiliations:** ^1^Jade University of Applied Sciences, Section Technology and Health for Humans, Oldenburg, Germany; ^2^Leibniz Institute for Prevention Research and Epidemiology - BIPS, Bremen, Germany; ^3^University of Bremen, Health Sciences Bremen, Bremen, Germany

**Keywords:** recruitment, older people, hard-to-reach people, participation, technology development

## Abstract

**Introduction:**

The participation of older people in research and development processes has long been called for but has not been sufficiently put into practice. In addition, participation is often late and not particularly intensive, so that certain older groups of people are underrepresented in the development of health technologies (HT). Heterogeneity, e.g., between urban and rural populations, in access to and motivation for participation is also rarely taken into account. The aim of this study was to investigate form and phases of participation for hard-to-reach older people in the research and development process of HT.

**Methods:**

The qualitative study among multipliers was conducted using focus groups and telephone interviews and took place in a city and an adjacent rural area in northwestern Lower Saxony, Germany. A content analysis of the data was undertaken using deductive-inductive category formation.

**Results:**

Seventeen participants (13 female) took part in the study (median age 61, 33–73). Participants from both areas identified particular forms and phases of participation in the research and development process. Longer forms of participation for hard-to-reach groups and the development process of technologies for older people from the rural area were viewed as challenges. Passive and active access strategies are needed to achieve sufficient heterogeneity in the research and development process. Trusted multipliers can play an important role in gaining access to hard-to-reach older people, but also during the research process. Apart from facilitating factors (e.g., age-specific study materials), inhibiting factors such as contact anxieties are also indicated. Only urban participants mention financial/material incentives and community as possible motivations.

**Conclusions:**

The results provide important insights from the perspective of multipliers. They show specificities in access and participation for rural areas and for hard-to-reach older people. Many older people may have uncertainties about research projects and HT. Multipliers can assume a key role to help reduce these uncertainties in the future.

## 1 Introduction

### 1.1 Participation formats for older people

Due to existing and increasing heterogeneity, the participation of older people in the research and development of technologies is becoming more and more important having been long called for (1). Involving older people in the research process can, among other things, increase the relevance of research questions, facilitate access to other older people, and lead to improved clarity and quality of study materials. Apart from a better understanding of the data collected, collaboration can also promote a better relationship between the actors and older people's acquisition of competencies (2, 3). Participation in the development process can help to improve design and user-friendliness (4, 5).

In this context, participatory health research takes on particular significance. Its key feature is the direct involvement of people whose working or living conditions lie in the focus of the research. The people being studied are understood here as partners in the research who, as much as possible, are involved on an equal footing in the conception and implementation of all phases of the research process, from selection of the research topic to the interpretation of the results (6). According to Chung and Lounsbury (7), participation takes four forms: compliant participation in studies (e.g., interviews) as the simplest form, directed consultation (e.g., expert interviews), and mutual consultation (e.g., project advisory boards), with empowering co-investigation as the highest form. A variety of models are available for participation in technology development, among which human-centered design (8) is the most frequently used. The development process is iterative, and consists of four phases: analysis of the context of use, determination of the requirements for use, drafting of, and evaluation of, the design solutions.

Although these approaches and models have been known for years, their translation into practice in the research and development process has thus far been inadequate (1, 9). The reasons given for this include insufficient financial resources and time, but also researchers' lack of knowledge in the field of participatory research (9–12). In addition to assisting with recruitment, Kylén et al. report (2) that older people were most often involved as members of advisory boards, or helped in drafting the study design, or in formulating research questions. A survey among older people (13) indicated that most respondents would choose the compliant participation in studies (e.g., interviews), but they could also envision more active collaboration in the research process, e.g., in defining the research questions.

Many studies show that older people were often not involved in the development process of HT until the evaluation stage, and participation took place largely at a low- to medium level (5, 12, 14). In their review, Fischer et al. (5) report that older people were most often involved in information gathering, device testing, or advising. Collaboration by way of a participatory design only occurred in three of the 40 studies evaluated. Another study (13) on older people's ideas revealed a mixed picture regarding the degree of involvement. It was important for some participants to take part in all four phases from analysis of the context of use, to evaluation of the design solutions. Other participants could only envision participating in individual phases. The main reasons given by those who could not envision participating were lack of time and competencies, and the complexity of the subject.

To develop age-specific technologies, individual competencies, the needs (15, 16) and the increasing heterogeneity of old age should already be taken into account early in the development process (17). Current research shows that the older people who participate are often younger, healthier, and have a higher level of education, or have study experience (5, 13, 15–19). Certain groups of older people are thus underrepresented in the research and development process, and the targeted user group is insufficiently represented (12, 16, 20). Even though old age is characterized by its heterogeneity, it is not uncommon for older people to be described as a rather homogeneous group (5, 12, 21). It is striking how often data on the level of education, technical competence, and cultural background are missing (21, 22), even though these characteristics are often associated with older peoples' access to and use of technology (22, 23). Not considering these characteristics creates the danger of overlooking individual needs, distorting results, and thus of drawing false conclusions regarding technology development, thus exacerbating digital inequalities (16, 17, 24). The place of residence can also have an influence on older people's use of technologies (25). Older people living in rural areas are more likely to benefit from such technologies (e.g., assistive technologies, telemedicine (26)).

### 1.2 Strategies in access and participation of older people

Gaining access to older people in the research and development process can be a challenge (16, 20, 27). Due to lower population density in rural areas, and their geographical location, recruiting older rural people can require a greater commitment of logistical, time, and financial resources (28). Rural areas are characterized by additional challenges in terms of digitalization and technology use. European surveys (29) report that the likelihood of older respondents using the Internet is lower in rural than urban areas, possibly due to lack or insufficient development of infrastructure (30) or access (31) to the Internet. In addition, demographic change in rural areas is leading to further challenges in healthcare provision. A greater prevalence of various chronic disorders in the rural population (e.g., obesity (32), diabetes mellitus type-2, and coronary heart disease (33, 34)) has been reported.

Numerous studies have been published about strategies and factors in older people's access to, and participation in, the research and development process. A distinction can be made between passive and active strategies (35–37). Passive strategies include, for example, media, printed material (e.g., brochures, flyers), letters, and telephone calls (13, 27, 38–40). In addition, social media have recently become more and more important for recruitment (41–43). Thus, more studies are using this method exclusively, or in combination with other strategies, to gain access to different groups of people.

Certain older groups of people (e.g., people from a migrant background) often require a targeted approach, since passive strategies are insufficient to motivate them to participate (27). Apart from approaches involving the municipality, approaches derived from participatory action research and community-based participatory research are being increasingly used to gain access to hard-to-reach people (9, 44). Multipliers have a special role to play in gaining direct access to people. They can be from a particular professional group in the healthcare system (e.g., medical staff), or in social work, or be themselves members of the group sought (e.g., be from a migrant background) (45). Collaboration between multipliers and universities in research can range from providing information about the project as the lowest level, to co-management as the highest level (46). According to a review of 66 studies, close collaboration in recruitment took place in 21 studies, and multipliers assumed a leading role in this connection. A longer-term, equal partnership took place in six studies (47). In the context of collaboration in the field of technology development, different institutions and multipliers were involved in gaining access to older people (e.g., seniors'- and church organizations) (5, 12, 38). However, it often remains unclear which older groups of people were thus reached, and how effective these modes of access were in relation to technology development. The description of people who are hard-to-reach for the research and development process is currently not internationally uniform. Although the term is increasingly the target of criticism (48), it will be used in this study, as in others (49), to describe groups of people whose participation poses particular challenges due to their physical, geographic, socioeconomic, or sociocultural situation.

### 1.3 Factors influencing access and participation of older people

Access to older people can be influenced by facilitating and inhibiting factors. Factors that are conducive to participation in studies include provision of transport (40), easily accessible informational and study materials, an open, transparent and respectful approach to potential participants (48–50), and both material and immaterial incentives (40, 50). In dealing with older and hard-to-reach people, the research process should be flexibly designed (49, 50). Familiar, trusted people are also mentioned as conducive to gaining access (40, 48, 50). For the successful recruitment of older people from migrant backgrounds, being personally approached by multipliers can be particularly crucial (38, 48). Several studies (40, 48–51) emphasize that access means a long-term commitment, and collaboration involves intensive work. Apart from building trust vis-à-vis institutions and participants, additional resources (time, personnel, and financial resources) are emphasized as being keys to success. In rural areas specifically, additional resources should be made available, to be able to take into account the extended recruitment period, data collection, and rural particularities (28). Potential barriers include, for example, inaccessible informational and study materials, linguistic barriers, skepticism and mistrust of the research, personal constraints (e.g., health issues), but also lack of self-confidence (40, 49, 50).

### 1.4 Motivational reasons of older people

Older people can have different motivations for taking part in a study. Apart from its scientific usefulness, in various studies, personal reasons (such as curiosity, interest in the subject, trust), self-interest (among other things, promoting healthy aging) or usefulness to others were mentioned (19, 52). In a long-term study with older people in focus groups, the experience, social exchange and prior experience of other study participants were also mentioned as factors motivating to participate (13).

Despite the aforementioned call to involve older people more actively in the development process of HT (1), there is still a lack of approaches and strategies for hard-to-reach older people who have hitherto been particularly affected by inequalities and could benefit from these technologies. Above all, the perspective of multipliers from the context of older people, such as from community and senior services, can be helpful in developing addressee-specific access strategies for the research and development process in the future. To date, there is a specific lack of information contrasting rural and urban areas, and the gain to be achieved through combining participation options.

### 1.5 Objective

Against this background, the objective of this study was to investigate for hard-to-reach older people the form and phases of participation in the research and development process of HT. In addition, strategies, influencing factors, and motivations in obtaining access and for participation were studied. To this end, multipliers from community and senior services in urban and rural area were interviewed, and their experiences and ideas were compared.

## 2 Methods and methods

### 2.1 Design and context

The study was conducted with a qualitative design using digital focus groups and telephone interviews. We chose this design to be able to capture and compare participants' different experiences and perspectives. The project was part of the TECHNOLOGY subproject of the prevention research network “AEQUIPA” (Physical activity and health equity: primary prevention for healthy aging). The overall aim was to investigate and develop new technologies for individual preventive healthcare. The network's main topics included promoting physical activity among people over 65, use of new technologies in preventive healthcare, and health equity (53).

### 2.2 Recruitment and access

The sample included persons who are often in contact with different older people in their daily working lives, and who work in community and senior services in urban or rural areas in northwestern Lower Saxony. To select suitable participants, a qualitative sampling plan was initially defined, which provided for the division of the total into four different areas by urban/rural status. The city of Oldenburg and the adjacent rural areas of Oldenburg were chosen as locations for this study. The city of Oldenburg is surrounded by various rural areas (54). To define the relevant areas, we used the three levels of social space as a theoretical framework (55), which, apart from the individual's own home as a socio-spatial center, assigns different areas to the proximate social space (e.g., social contacts, action space) and the socio-spatial periphery (e.g., leisure, culture). In a second step, the areas were grouped into four categories including “social club/leisure” (e.g., neighborhood social clubs, cultural centers, multigenerational centers), “counseling/information” (e.g., senior counseling centers, neighborhood social clubs, cultural centers), “getting-involved/participation” (e.g., senior associations, civic associations), and “everyday support services” (e.g., neighborhood assistance, shopping and household assistance, professional or volunteer companion services). Service providers from the healthcare system (e.g., caregiver services, doctor's offices) were excluded. The qualitative sampling plan foresaw recruiting eight participants per area.

Potential participants were identified by doing a computer-assisted desktop search, and were then assigned to the four defined categories. In addition, contacts were asked to suggest other possible contacts for the respective categories. Invitations to participate in the study were sent to the people thus identified by email and/or they were contacted by telephone. If interested, an appointment was made, and participants received written information and a consent form informing them about the study and about data protection. The study was approved by the Research Ethics Committee of the Carl von Ossietzky University of Oldenburg (Drs. EK/2020/046).

### 2.3 Methods and instruments

Due to the Corona pandemic, the focus groups had to be conducted digitally. Alternately, telephone interviews were offered to participants who were unable to take part in the focus groups. The telephone interviews and digitally conducted focus groups were structured and guided. The guide included a schedule that arranged the questions into five areas: intro (groups of people with whom the participants are in contact in their work, experience with research and technology), interest and motivation (motivations of hard-to-reach older people, subject areas), modes of access (strategies, influencing factors for the access of hard-to-reach older people), participation (forms of participation, involvement in research and development process), and conclusion (other topics). From start to finish, the focus groups were supported by a PowerPoint presentation in which pictures with examples (technologies in the field of prevention using the example of AEQUIPA (4, 39), participation models (7, 8, 56)) were shown as visual stimuli. In addition to the guide, participants were sent in advance a brief questionnaire containing details about the person (age, sex, area) and field of activity (institution, responsibilities and field of work). Prior to the focus groups/interviews, pretests were conducted with two people from two different institutions (neighborhood social club, civic association) using the think-aloud method (57). The focus was on the comprehensibility and the sequence of the questions asked.

### 2.4 Data collection

The telephone interviews and focus groups were and carried out by an experienced health scientist and recorded. Characteristics and the course of the discussion were documented during the sessions and phone calls. Each of the two focus groups lasted between 120 and 140 min, and each of the two telephone interviews between 30–45 min. Prior to the focus groups, the participants received a link to the video conferencing system. Eight participants took part in the “city” group session and six participants in the “rural areas” group session. Telephone interviews had to be conducted with three participants, two of whom took part in a single call at the same time. To ensure comparability with the focus groups, before the calls, participants received documents for the purpose of visualization, analogous to the presentation in the focus groups with the technologies in the field of prevention, and using the AEQUIPA example (4, 39) and the models on the subject of “participation” (7, 8, 56).

### 2.5 Data analysis

The analysis of the transcripts was carried out using content-structuring qualitative content analysis per Kuckartz (58). Category formation was carried out using a computer-assisted deductive-inductive method with MAXQDA version 2022, and using the seven phases described by Kuckartz (58). The deductive categories were based on the aforementioned guide. In addition, relevant passages in the material were inductively coded, and further categories were formed. Like the guide, the code system is comprised of 11 categories with assigned subcategories, in which three groups (urban, rural, urban+rural) were distinguished and compared to one another. The urban+rural group included two participants, who were responsible for both areas and could not be assigned to either of the two groups. The coding of the material was carried out by one person. The coding guide, codes, and anchor citations were then checked by a second person, and discussed by the two researchers. The questionnaires were descriptively analyzed using SPSS version 23.

## 3 Results

### 3.1 Sample description

Table 1 shows the sample of 17 participants (13 female), of which 14 participants took part in a focus group. The median age at the time of the study was 61 (33-73). A telephone interview was conducted with three participants. In both areas, responsibilities and fields of work consisted mainly of counseling, information, and relaying with respect to various subjects (e.g., services for older people) (13 mentions) and organization, coordination, and carrying out of services, projects and events (12 mentions) for a variety of older people. The facilities were predominantly operated by non-profit organizations. No private operators were mentioned.

**Table 1:** Sample description.

**Characteristics**	**Urban**	**Rural**	**Total**
Participant (female): *n*	8 (6)	9 (7)	17 (13)
Age: Md (min–max)	61.0 (33-73)	63.0 (46-73)	61.0 (33-73)
Area of responsibility: *n* (MR)^*^	10	9	20
**Carrier of the facility:** ***n*** (MR)^*^			
Public carrier	3	3	6
Non-profit carrier	6	6	12
Private carriers	/	/	/
Other carrier	1	3	4
**Tasks/activities:** ***n*** (MR)^*^			
Accompaniment/ care/support	6	3	9
Counseling/ information/ relaying	8	5	13
Networking/ public relations	6	2	8
Organization/coordination/carrying out	6	6	12
Training	4	2	6
Representation of interests	2	1	3
Administration/committee work	2	3	5

^*^Multiple response (MR) was possible (n = 2).

Urban participants report that they mostly deal with people aged 55–80 years. Most of the older people in the courses and counseling services were female. According to the participants, the older people often come from precarious situations and are in need of assistance. Two facilities offer services specifically for older people from migrant backgrounds. According to the participants from rural area, the people in their facilities are more than 60 years old. They are often older people who still live alone at home with assistance, are partly in need of care, have little social contact, and whose physical activity is limited. Particular group meetings were held for older people suffering from dementia and for nursing home residents. According to the participants, facilities in rural areas have little contact with older people from migrant backgrounds. In the case of the two participants responsible for both areas, the people included persons over 65, some of whom have physical disabilities. Specific services are offered for persons suffering from dementia, and for older people with disabilities.

### 3.2 Participation formats for older people

It was possible to derive three subcategories (concerns/obstacles, individual forms/phases, all forms/phases) for the forms of participation (7), for the research process (56), and for the development process (8) (Table 2). Participants from both areas mentioned the long duration of an empowering co-investigation through a participatory study as “concerns/obstacles” to participation. Some also noted that participation never managed to reach large groups of older people, and only people who are younger and more active took part. The following quote underscores this point: “*If they're still somewhat younger, then maybe, but the older ones no, I don't think so”* (TI_urban+rural, pos. 70). As for the other forms, participants from both areas regarded directed consultation as appropriate, since it identifies the older person as an expert. Participants from the rural area could also envision the compliant participation in studies. In the opinion of participants who were responsible for both areas, mutual consulting appeals to older people for whom the subject is relevant. Participants view as realistic a one-time participation of older people from migrant backgrounds with reference persons. An empowering co-investigation through a participatory study is seen as more appealing for younger, and more active older people. Individual participants from both areas stressed that the best-suited older people need to be found for all forms.

**Table 2:** Participation formats^a^: (69 coded text segments).

**Subcategories (coded text segments)**	**Urban**	**Rural**	**Urban/rural**
**Participation formats**^b^ **(23)**			
Concerns/obstacles (8)	1	5	2
Individual forms (11)	2	7	2
All forms (4)	1	3	/
**Participation phases—research process**^c^ **(13)**			
Concerns/obstacles (4)	/	3	1
Individual phases (7)	1	2	4
All phases (2)	1	1	/
**Participation—development process technologies**^d^ **(33)**			
Concerns/obstacles (8)	/	5	3
Individual phases (10)	3	3	4
All phases (14)	5	8	1

^a^Assigned text segments from the transcripts of the focus groups (FG) and telephone interviews (TI).

^b^Participation according to Chung and Lounsbury (7) using four forms: compliant participation in studies, directed consultation, mutual consultation, and empowering co-investigation.

^c^Participation phases in the research process is based on the definition of von Unger (56).

^d^Human-centered design (8) is based on four phases: analysis of the context of use, determination of the requirements for use, drafting of design solutions, and evaluation of the design solutions.

Participants from the rural area viewed the fact that it is difficult to find hard-to-reach older people as “concerns/obstacles” for participation in the research process. It takes a lot of time to do this, and it is only possible with support from existing structures. As for individual phases in the research process, urban participants mentioned the presentation of results by older people from the community. The following quote from a rural participant describes another phase: “*(...) they would (...) perhaps also (...) probably take part in the development of a questionnaire (...)”* (TI_rural, pos. 44). Participants responsible for both areas mentioned data analysis as a possible phase for older people who have particular functions or roles within a community. Individual participants from both areas regarded participation in all phases as more realistic for younger and more active older people.

Participants from the rural area, and those who were responsible for both areas, saw fundamental challenges with respect to involving older people from the rural area in the development process of HT. Participation would only be possible if there were easy access, and the phases were flexibly designed and planned in small packets. Some participants in both areas could envision older people both in individual phases and, under certain circumstances, in the complete process. In the view of urban participants, there are better chances of finding older people for the analysis of the context of use and the conception of design solutions. In the opinion of other participants, evaluating design solutions is easiest for many older people, since they can directly try out the technology developed. It was suggested that older people could play a role in different tasks and in the development process. To get older people involved in the whole development process, participants from the rural area thought that recruitment of older people should be done in collaboration with multipliers. Urban participants also believe that older people can be successfully recruited: “(...*) I can imagine that you can also really find people for the different areas (...)”* (FG_urban, pos. 157).

### 3.3 Strategies in access and participation of older people

Three passive and three active strategies were derived from the interviews (Table 3). Participants from both areas mentioned mainly printed materials and media as a passive strategy. But they also stressed that it is difficult to make contact with hard-to-reach older people using this strategy, since only those who are otherwise interested and active get in touch. The following quote highlights a problem with printed materials: “*(...) if you put flyers down somewhere (...), they lie there and nobody takes them. But it's another matter if I'm standing somewhere and can also tell people a story about it and then hand them a flyer (...)”* (FG_rural, pos. 94). In addition, participants from the rural area stressed that older people with limited financial resources are excluded from media, since they often cannot afford newspapers. Participants from the rural area stressed the great importance of word-of-mouth publicity, especially in rural areas.

**Table 3:** Strategies in access and participation^*^: (69 coded text segments).

**Subcategories (coded text segments)**	**Urban**	**Rural**	**Urban/rural**
**Passive strategies (21)**			
Word-of-mouth publicity (5)	/	4	1
Printed materials (5)	3	1	1
Media/press relations (11)	5	5	1
**Active strategies (48)**			
Personal approach to specific places (14)	5	8	1
Personal, written/telephone address (2)	/	/	2
Addressing through reference persons, multipliers (32)	12	17	3

^*^Assigned text segments from the transcripts of the focus groups (FG) and telephone interviews (TI).

In the view of participants from both areas, the active strategies included approaching older people via reference persons and multipliers, as well as personally approaching them in particular venues (e.g., in senior groups). They suggested first informing the multipliers about the subject, and the latter can then provide support in recruiting older people. An urban participant mentioned an example of this for approaching older people from migrant backgrounds: “*So someone from their own culture would really have to go there, talk to them, and bring them along”* (FG_urban, pos. 24). In the view of participants from the rural area, both passive and active strategies are needed to achieve sufficient heterogeneity among the older people. However, active strategies are of greater importance for recruiting. Participants emphasized that successful access and involvement could only be ensured if the older people trust the interlocutor, but that this trust had first to be established. Participants responsible for both areas also mentioned approaching older people in writing or by telephone, if there are already contacts.

### 3.4 Factors influencing access and participation of older people

Seven subcategories of facilitating factors and five subcategories of inhibiting factors were derived (Table 4). In the opinion of participants from both areas, trusted reference persons and multipliers were facilitating factors. For older people from migrant backgrounds, it could be helpful if people from the same cultural background also took part. Certain characteristics of the researcher (e.g., same cultural background, same gender or age) can also have a positive impact. Sufficient time for developing a relationship and building trust should also be allowed for, as the following quote makes clear: “*(...) especially in this technical field, to help people who have had few points of contact with it to learn about it, I think the duration of such projects should be a minimum of five years (...)”* (FG_rural, pos. 70). All participants stressed the importance of accessible speech and writing, and of attitudes toward older people. Communication should be simple, patient, and understandable. Study materials should be formulated in a variety of languages. The setting should be accessible too, such as easy-to-reach places with transport services.

**Table 4:** Influencing factors^*^: (75 coded text segments).

**Subcategories (coded text segments)**	**Urban**	**Rural**	**Urban/rural**
**Facilitating factors (21)**			
Memories (2)	/	2	/
Homogeneous groups (2)	1	1	/
Trust (5)	2	2	1
Time (5)	2	3	/
Accessibility: easy-to-reach places, transport services (6)	1	4	1
Accessibility: language, writing, attitude (20)	9	9	2
Characteristics of the researchers (5)	3	1	1
**Inhibiting factors (30)**			
High effort/high forms of participation (12)	/	6	6
No accessible language, writing, attitude (4)	2	1	1
Personal impairments (1)	/	1	/
Lack of/willingness to participate/interest (6)	/	6	/
Contact anxiety (7)	2	3	2

^*^Assigned text segments from the transcripts of the focus groups (FG) and telephone interviews (TI).

In the view of participants from both areas, speech, writing and attitudes that are not addressee-specific represented an obstacle. There could also be anxieties about having contact with the researcher or other older people: “*(...) this is something else that should not be neglected in the case of people from migrant backgrounds (...) that there are often (...) very great inhibitions about going to groups somewhere where there are only German seniors”* (FG_urban, pos. 86). Participants from the rural area and those responsible for both areas stressed that requiring too much from the older people, and higher forms of participation, could be inhibiting. According to individual participants from the rural area, vision, hearing, or mobility problems could inhibit participation. Participants from the rural area mention a lack of interest, or of willingness to participate, as compared to the city. Rural areas are a world of their own, with entrenched structures and traditions, in which it could be more difficult to find suitable older people: “*(...) they have their garden, they have their house (...). They simply have their own little world, (...) in the countryside there is still order. (...) And you hardly have a chance with newer things (...)”* (TI_rural, pos. 16). In the opinion of the participants, older people from these areas often have fewer points of contact with technical innovations.

### 3.5 Motivations of older people

Four subcategories were derived for this subject (Table 5). Participants from both areas mentioned interest, curiosity, and personal benefit as motivations for older people to participate. The subject should be relevant and part of the normal world of the older people. Prior experience and trust in reference persons and multipliers who, for example, run events, or accompany participants, were also mentioned. Urban participants mentioned financial or material incentives, particularly for older people who have little money: “*(...) they need a reward somehow, (...) some form of profit (...) and not just that they get to be involved and yes, thank you, but whether it's compensation in the form of money or a course (...), something material (...)”* (FG_urban, pos. 60). Other participants emphasize that many older people are less interested in a financial incentive than in an exchange in a familiar setting, or community, that meets anyway and could also be jointly involved in a study.

**Table 5:** Motivation to participate^*^: (35 coded text segments).

**Subcategories (coded text segments)**	**Urban**	**Rural**	**Urban/rural**
Previous experience, trust (6)	4	/	2
Exchange, familiar environment, community (5)	5	/	/
Interest, curiosity, own benefit (19)	7	5	7
Financial/material incentive (5)	5	/	/

^*^Assigned text segments from the transcripts of the focus groups (FG) and telephone interviews (TI).

## 4 Discussion

This qualitative study analyzed forms and phases of participation, strategies, influencing factors and motivations in the access to, and participation, of hard-to-reach older people in the research and development process of HT. Differences from the perspective of multipliers from the community and senior services in the city and in a rural area were compared.

The predefined qualitative sampling plan exhibited sufficient saturation in the areas. It was not possible, however, as originally planned, to distinguish between the proximate social space and the socio-spatial periphery, since the search for suitable participants in rural areas proved to be difficult. Queries sent to civic associations or church organizations in this area often went unanswered. One explanation could be that urban establishments are more used to receiving inquiries about research projects. Another explanation could be the structures and services of the city, which are, despite the geographical proximity, more diverse compared to the rural area. The two areas differ in the specific services available for people from migrant backgrounds. The Corona pandemic may also have led to this reluctance. Participants' suggestions about other institutions and people from the rural area led to three additional interviews.

### 4.1 Participation formats for older people

The discussions with participants from both areas made clear that older people are more susceptible to becoming involved in individual forms and phases than in the complete research and development process. According to the participants and current research practice (5, 13, 15–19), younger and more active older people are more likely to take part in higher forms of participation (7) like empowering co-investigation using a participatory study. For a participatory study within the context of the research process, different phases, from questionnaire development to presentation of the results, were indicated by older people, which differs from the results of another study (13). In the latter case, however, older people were surveyed using questionnaires. Despite a positive assessment of collaboration, it is a challenge, especially in rural areas, to find suitable older people for the development process of new HT. Hence, participation can only succeed if multipliers are involved in recruitment, and the phases are designed to be easily accessible and flexible. In particular, concepts for the participation of hard-to-reach older people in the development process of HT have been lacking. The current findings provide important information in this regard. As in current research practice (5, 14), participation at the beginning of the development process to gather information, and during the evaluation of technologies, was mentioned the most. After participation in all phases in the development process was recommended (1), the participants from the city discussed how to involve hard-to-reach older people using different tasks and roles. James and Buffel (3) recommended offering participants a flexible framework in the research process, from which they can choose responsibilities and roles. Older people could be differently involved in the development process (11). To do justice to increasing heterogeneity, structures for different groups of people should be created for this purpose, taking into account the fact that an older person's form of participation may change over the course of a project from strong participation to none (3). Although this study does not discuss methods, the results from the study and from the viewpoint of older people suggest (13) that homogeneous group sessions in a familiar setting are especially acceptable solutions for hard-to-reach older people.

### 4.2 Strategies in access and participation of older people

In terms of strategies to gain access to hard-to-reach older people, the participants from both areas mostly emphasized approaching older people via reference persons and multipliers (e.g., from migrant backgrounds, from rural areas) as an active strategy, and thus confirmed prior reports (5, 12, 13, 27, 40, 48, 51). It is notable that the participants in this study see themselves taking a more active role in this subject in the future. It was thus suggested, for instance, that multipliers could disseminate study information in an addressee-specific manner, and identify potential individuals. The concrete role of collaboration was not addressed in this study, however, but will be taken up in future studies. Wieland et al. (47) suggest that closer collaboration, or a longer-term partnership with multipliers on an equal footing, improves access. To achieve sufficient heterogeneity, some participants in this study recommended using passive and active strategies, which confirms experience from a study in a rural area (51). However, in contrast to the current study, the focus was not only on older people and not on HT. Our results therefore fill an important research gap. However, the strategies mentioned can certainly also be helpful for other non-technology-orientated research topics. Although printed materials, for example, can have a positive impact on recruitment as a passive strategy (39, 42), they were only assigned a subordinate role in gaining access to hard-to-reach older people in this study. Bajraktari et al. (42) report, for example, that by mailing materials, they were able to recruit almost 50% of their older people for a study on digital fall prevention. The older study participants were, however, predominantly female, tech-savvy, and had a higher level of education. In terms of the use of media, participants from the rural area noted that in particular rural older people may not be reached by printed announcements, such as newspapers, because access is limited. This aspect is of great significance for avoiding inequalities, and should be taken into account in planning the study. Interestingly, participants did not mention social media as a complementary passive strategy. Nonetheless, in the future, this method could represent a valuable addition to the traditional strategies, especially in recruiting older people in rural areas and hard-to-reach groups of people (28, 41–43). It should be kept in mind, however, that the user-friendliness of social media platforms can represent a barrier precisely for older people, since they are often tailored to younger people (43).

### 4.3 Factors influencing access and participation of older people

Consistent with research practice, participants from both areas in this study underscored the great importance of an easily accessible study plan (e.g., study materials, event venue) (40, 48–50). Especially when dealing with older people from migrant backgrounds, cultural particularities need to be integrated into the plan (e.g., people from the same cultural background). In this context, Acha et al. (49) found that, apart from competencies in the field of participatory research, researchers' knowledge of cultural particularities is a facilitating factor. In addition, some participants from both areas stressed that building trust with multipliers and older people requires a lot of time and that more relevant resources need to be included in the plan. A flexible design of the research process, including sufficient time, was thus recommended (40, 49, 50), which was also clearly highlighted in the current study. Some participants from the rural area emphasized this, particularly in the context of studies on HT. Especially in rural areas, some older people seem to have a fear of contact. Only participants from the rural area mentioned that a too-great time investment, and more complex forms of participation as inhibiting factors, which confirms findings on barriers for older people (13). According to such findings, however, contact anxieties can also be inhibiting (40, 49, 50); these anxieties can be addressed by the methods suggested by the participants, such as, for example, forming homogeneous groups and choosing suitable characteristics of the researcher (cultural background, sex).

### 4.4 Motivational reasons of older people

The motivations mentioned by participants confirm previous findings (13, 19, 52). Financial incentives were also often discussed as a motivating factor (39, 48, 50), but only by a few urban participants. Precisely for hard-to-reach older people who meet regularly, the community can have greater added value by virtue of social solidarity, and the protective and familiar setting. It has already possible to acquire this sort of experience with preventive technologies in dealing with older people from migrant backgrounds (13, 38).

## 5 Strengths and limitations

To the authors' knowledge, this is the first qualitative study on technology-research participation from Germany that has looked at the perspective of urban and rural multipliers from community and senior services. Its strengths include the involvement of participants from different institutions in two areas. The perspective of participants from the rural area indicated the challenges of working with older adults who experience inequality-inducing factors (e.g., income, experience with technology), which can also have an influence on access and participation. Another strength is the participation of two persons from a seniors' advisory board from each of the two areas, who could thus represent their age group. In contrast to other studies, the focus in this study was on analyzing different hard-to-reach older people and their possibilities for participation using three different models. The findings thus complement current research in the field of participatory research. Although various models of participation were discussed, the study does not address the methods that might be used during the participation. In the same way, the forms of possible collaboration with multipliers were not discussed with the participants. These aspects should be further investigated in future studies. In addition, the results only relate to two defined areas as perceived by 17 participants. Generalisability to other areas is only possible to a limited extent, as other structures and services are presumably available there and the participants have different experiences in dealing with older people. Nevertheless, the findings from the perspective of participants who are in contact with older people on a daily basis provide important insights for future research projects. Although a variety of people from community and senior services participated, the perspective of people from the healthcare system (e.g., doctors, nursing staff) was not considered. This perspective could be particularly important for rural areas and should be taken into account in planning future studies. As participatory research in HT development for older people gains traction, their involvement in all phases gives participatory research great importance for proactively minimizing expected inequalities in the access to and use of HT.

## 6 Conclusions

The results from the qualitative study illustrate that, especially in rural areas, access to older people, but also to multipliers, requires more time for establishing contacts and building trust for participation in research and development processes. In comparative terms, older people from rural areas have other interests and are shaped by different traditions, which can have an impact on motivation and strategies in gaining access for participation. Possible uncertainties vis-à-vis research projects and HT are additional factors. To reduce these, the multipliers envision that they could take an active role in the future. It should be noted that in particular the place of residence can be an important characteristic that, in combination with other characteristics (e.g., gender, cultural background), can further exacerbate digital inequalities. In addition, the results of this study suggest that only a proportion of older people want to be involved at the highest level over an extended period of time. To nevertheless be able to map the different needs, researchers should work together with multipliers to define flexible structures and processes from which interested older people can select the tasks and roles that suit them. For example, older people with a migrant background could be involved together with multipliers and in groups that are as homogeneous as possible. These challenges and the strategies derived for these can help future researchers to reduce digital inequalities, and to ensure the inclusion of diverse groups of older people in the health-technology development process.

## Data availability statement

The original contributions presented in the study are included in the article/supplementary material, further inquiries can be directed to the corresponding author.

## Ethics statement

The studies involving humans were approved by Commission for Research Assessment and Ethics at Carl von Ossietzky University Oldenburg prior to implementation (Drs. EK/2020/046). The studies were conducted in accordance with the local legislation and institutional requirements. Written consent of the participants or their legal guardians was required. The patients/participants or the patients'/participants' legal guardian(s)/next of kin provided their written informed consent to participate in this study.

## Author contributions

AP: Conceptualization, Data curation, Formal analysis, Investigation, Methodology, Project administration, Resources, Supervision, Validation, Visualization, Writing – original draft, Writing – review & editing. FK: Conceptualization, Funding acquisition, Writing – review & editing. HZ: Conceptualization, Funding acquisition, Writing – review & editing.
